# The Maritime Declaration of Health (MDH) as a tool to detect maritime traffic-related health risks: analysis of MDH forms submitted to Spanish ports, October 2014 to March 2015

**DOI:** 10.2807/1560-7917.ES.2017.22.24.30551

**Published:** 2017-06-15

**Authors:** Rosa M López-Gigosos, Marina Segura, Rosa M Díez-Díaz, Isabel Ureña, David Urzay, Patricia Guillot, Ana Guerra-Neira, Almudena Rivera, Ángeles Pérez-Cobaleda, Ascensión Martín, María Nuñez-Torrón, Begoña Alvarez, Mar Faraco, José M Barrera, María J Calvo, José Gallegos, Antonio Bermejo, Carmen Aramburu, Miguel Dávila, Fernando Carreras, Rosemarie Neipp, Alberto Mariscal

**Affiliations:** 1 Medical service, Port Health Authority of Málaga, Málaga, Spain; 2 Medical service, Port Health Authority of Tarragona, Tarragona, Spain; 3 Medical service, Port Health Authority of Tenerife, Santa Cruz de Tenerife, Spain; 4 Medical service, Port Health Authority of A Coruña, A Coruña, Spain; 5 Medical service, Port Health Authority of Vigo, Vigo, Spain; 6 Medical service, Port Health Authority of Huelva, Huelva, Spain; 7 Medical service, Port Health Authority of Santander, Santander, Spain; 8 Medical service, Port Health Authority of Algeciras, Algeciras, Spain; 9 Medical service, Port Health Authority of Barcelona, Barcelona, Spain; 10 Subdirección General de Sanidad Exterior, Ministry of Health, Madrid, Spain; 11 Department of Public Health, University of Málaga, Málaga, Spain

**Keywords:** International Health Regulation 2005, Maritime Declaration of Health, international travel, surveillance

## Abstract

The international maritime traffic of people and goods has often contributed to the spread of pathogens affecting public health. The Maritime Declaration of Health (MDH), according to the International Health Regulations (IHR) (2005), is a document containing data related to the state of health on board a ship during passage and on arrival at port. It is a useful tool for early detection of public health risks. The main objective of our study was to evaluate compliance with the model provided in the IHR, focusing on the format and degree of completion of MDH forms received at Spanish ports. We reviewed the content of 802 MDH forms submitted to nine Spanish ports between October 2014 and March 2015. Study results show that 22% of MDH forms presented did not comply with the recommended model and 39% were incomplete. The proportion of cargo ships with correct and complete MDH forms was lower than passenger ships; thus, the nine health questions were answered less frequently by cargo ships than passenger ships (63% vs 90%, p value < 0.001). The appropriate demand and usage of MDH forms by competent authorities should improve the quality of the document as a tool and improve risk assessment.

## Introduction

In today’s interdependent and interconnected world, it is possible for diseases to spread rapidly across the world and threaten public health. This is possible because of highly mobile human populations, international transport and trade. International public health security depends on the appropriate management of public health risks, which in turn depend on national capacities and international collaboration [1,2]. Effective public health measures and response capacities at airports, ports and ground crossings worldwide reduce the risk of the international spread of disease [3-5].

The International Health Regulations (IHR) (2005) [6] is a key international public health document that is legally binding across 196 countries, including all World Health Organization (WHO) Member States, requiring them to work together for global health security. This fundamental document requires that ratifying countries have the ability to detect, assess, report and respond to public health events [7,8].

The IHR (2005) includes provisions for the use of various health documents that can be presented, if requested, to health authorities on arrival at ports and airports [9]: the Ship Sanitation Certificate (Annex 3), the International Certificate of Vaccination and Prophylaxis (Annex 6), the Maritime Declaration of Health (MDH) (Annex 8) and the Health Part of the Aircraft General Declaration (Annex 9). If these documents are used inappropriately, it may result in an inadequate assessment of public health issues related to international traffic. There is also an IHR List of Authorized Ports to Issue Ship Sanitation Certificates, published and updated by the WHO, that lists all ports each state party has authorised to issue Ship Sanitation Certificates [10]. According to Article 37 of the IHR (2005) [6], ‘the master of a ship, before arrival at its first port of call in the territory of a State Party, shall ascertain the state of health on board, and, except when that State Party does not require it, the master shall, on arrival, or in advance of the vessel’s arrival if the vessel is so equipped and the State Party requires such advance delivery, complete and deliver to the competent authority for that port a Maritime Declaration of Health which shall be countersigned by the ship’s surgeon, if one is carried’. The state party may decide whether it requires all arriving ships to submit a MDH, which should conform to the model provided in Annex 8 of the IHR [6]. For European Union (EU) countries, according to Directive 2010/65/EU, the MDH is one of the forms that must be submitted to the first European port of arrival [11]. The EU SHIPSAN ACT Information System (SIS), developed within the scope of the EU SHIPSAN ACT Joint Action [12], allows port health authorities to confidentially share completed MDH forms between EU ports. Seamless and close communication between the master and port health authorities via the MDH enables the latter to detect on-board health problems that might constitute a risk to public health at an early stage and to take immediate measures to reduce the risk of onward transmission [13]. Therefore, it is essential that a MDH form is completed correctly at the onset of the assessment [13].

The International Maritime Organization (IMO) is a specialised agency of the United Nations that is responsible for measures to improve the safety and security of international shipping, and to prevent pollution from ships. One of its conventions, the Convention on Facilitation of International Maritime Traffic (FAL Convention), aims to facilitate maritime transport by simplifying and minimising formalities, data requirements and procedures associated with the arrival, stay and departure of ships engaged in international voyage [14]. The FAL Convention's main objectives are to ‘prevent unnecessary delays in maritime traffic, to aid co-operation between Governments, and to secure the highest practicable degree of uniformity in formalities and other procedures. In particular, the Convention reduces the number of declarations which can be required by public authorities’ [14]. At present, according to FAL Convention, ships may be required to complete nine documents: seven IMO-standardised forms (the General Declaration, Cargo Declaration, Ship's Stores Declaration, Crew's Effects Declaration, Crew List, Passenger List and Dangerous Goods Declaration), one Universal Postal Convention document and one International Health Regulations document (the MDH). Health authorities do not systematically inspect all ships, however, they review all the MDH presented.

Spain's geographical position, and the fact that it is a peninsula with 8,000 km of coastline with access to the Mediterranean Sea and the Atlantic Ocean, favour the frequent passage of ships [15]. As of 2016, Spain had 33 ports authorised to issue Ship Sanitation Certificates under the IHR [10]. All of these have medical inspectors (port health authorities) to evaluate events that suggest health risks on ships. For these reasons, and because there are numerous ports with high volumes of maritime traffic, cargo and passengers, Spain was considered ideal in terms of studying the quality of MDH forms.

The aim of this study was to examine current MDH practice, as there are no previous studies on this issue, in order to enable health authorities to improve their capacity to detect and respond to health problems on board ships. For this purpose, we were mainly interested in evaluating whether the MDH forms used by ships conformed to the model set out in Annex 8 of IHR (2005), the extent of the form’s completion by ships, and whether there were differences in completion and content depending on the type of ship or the geographical area of the port.

## Methods

### Study design

This was a descriptive study of MDH forms presented to Spanish ports by ships from all shipping routes. The object of analysis was the MDH form submitted by each ship to port authorities. The study analysed and compared MDH forms from different types of ships, as well as different MDH types.

### Sample size

We calculated the representative sample size using the following information: in 2014 ca 140,000 ships arrived at Spanish ports [15]. First, we studied MDH forms presented to port authorities over a 6-month period, from October 2014 to March 2015. Then, based on an estimated population of 70,000 ships over a 6-month period, with a 99% confidence level and a 5% margin of error, the calculated minimum sample size was 569 MDH forms.

### Participating ports

Nine of Spain’s 33 IHR-authorised ports participated in this study: A Coruña, Santander and Vigo (Atlantic Ocean ports on the northern coast); Algeciras, Santa Cruz de Tenerife and Huelva (Atlantic Ocean ports on the southern coasts and Canary Islands); and Barcelona, Malaga and Tarragona (Mediterranean Sea ports). They were selected based on geography (to obtain information on the different shipping routes) and their inclusion on the IHR list of ports authorised to issue Ship Sanitation Certificates at the time of the study [10]. We requested 100 consecutive MDH forms from each port from October 2014 onwards in the order in which they were presented at the port.

### Questionnaire to study MDH forms

Questions and items on the model MDH form (Annex 8 of IHR 2005) include [6]: (i) questions regarding general ship information such as port and date of submission, name of ship, registration/IMO number, port of origin and destination, flag of ship, master’s name, gross tonnage, port and date of issue of sanitation control certificate, inspection requirement, visit of affected areas identified by WHO (ports and date), and number of crew members and passengers on board; (ii) health-related questions regarding number of deaths on board due to illness, presence of infectious diseases on board during passage, frequency of ill crew and passengers, presence of ill persons at the time of MDH submission, medical consultation, presence of disease transmission risk factors, control measures taken, presence of stowaways on board, and presence of sick animals on board; (iii) signature of captain, signature of surgeon (if carried), and date of MDH . Finally, for each individual aboard the ship who is ill, the MDH is to be accompanied by a form called the Attachment to Model of MDH. This attachment requests information about the person’s age, sex, nationality, date they joined the ship, nature of illness, date of symptom onset, etc. 

Based on the model MDH form [6], we developed a questionnaire to assess the filling in completion and correctness of each field of the MDH forms used by the ships, which are based on but may differ from the model. The answers have been categorised into five options: (i) Yes, correct (field completed and correct); (ii) Yes, wrong (field completed but contains an error); (iii) Yes, unclear (field completed but illegible); (iv) No (field not completed); and (v) Not applicable.

Other questions included in the questionnaire concerned (i) the model of the MDH: based on the format shown in Annex 8 of the IHR (2005) or another format; (ii) the ship type: cargo ship or passenger ship; (iii) the port of submission: A Coruña, Santander, Vigo, Algeciras, Santa Cruz de Tenerife, Huelva, Barcelona, Málaga or Tarragona; (iv) the geographical location of the port: Mediterranean Sea coast, North Atlantic Ocean coast and South Atlantic Ocean coast (Table 1); (v) the ‘type’ of MDH: positive (health problem(s) on board) or negative (no health problem(s) on board); (vi) the validity of the Ship Sanitation Certificate: yes or no. Yes, if A. and B. below were correct; No, if A. or B. were not correct. A. Ship Sanitation Certificate issued by an authorised port i.e. one included in the IHR list of ports authorised to issue Ship Sanitation Certificates at the time of the study; and B. Ship Sanitation Certificate stated date of expedition less than or equal to 6 months according to Article 39 of the IHR (2005). This period may be extended by one month if the required inspection or control measures cannot be accomplished at the port.

**Table 1:** Number of analysed Maritime Declaration of Health forms from different ports of call, Spain, October 2014 to March 2015 (n = 802)

The ports and their geographical area	Number of MDH forms (n)	Percentage of total (%)
**Atlantic Ocean ports on the northern coast**	**209**	**26**
**A Coruña**	101	13
**Santander**	27	3
**Vigo**	81	10
**Atlantic Ocean ports on the southern coasts and the Canary Islands**	**285**	**36**
**Algeciras**	93	12
**Huelva**	95	12
**Santa Cruz de Tenerife**	97	12
**Mediterranean Sea ports**	**308**	**38**
**Barcelona**	100	12
**Tarragona**	101	13
**Málaga**	107	13
**Total number of MDH forms**	**802**	**100**

MDH: Maritime Declaration of Health.

To avoid inter-observer bias, all MDH forms were reviewed by a single observer. Any uncertainty of the single observer was resolved by the first four authors of the study via a specific procedure.

Data were entered and analysed using SPSS 15.0. We undertook descriptive statistics for each variable in the questionnaire. For those variables that could influence the distribution (e.g. type of ship, geographical area of the port and type of MDH (positive or negative), we used a chi-squared test to study the possible differences in the filling out of the questions on the MDH form in terms of completion and correctness. We studied the measures of association for nominal data: Pearson's contingency coefficient C, Cramer's V coefficient and phi coefficient. A value of p < 0.05 was considered statistically significant.

## Results

We analysed a total of 802 MDH forms submitted to nine selected ports. The ports and geographical areas where the MDH forms were submitted are shown in Table 1. Regarding these forms, 57% (n = 347) were submitted by passenger ships and 43% (n = 455) by cargo ships. Table 2 shows the proportion of each document field that was properly (completely and correctly filled), and the differences between cargo and passenger ships. We found that the nine health questions were properly answered in just 75% (n = 601) of the submitted MDH forms. The question asking whether the ship had visited an affected area was unanswered in 29% of MDH forms. In addition, 15% of the forms were not signed by the ship's master, which is required according to Article 37 of the IHR.

**Table 2:** Proportion of passenger and cargo ships with properly filled Maritime Declaration of Health forms, Spain, October 2014 to March 2015 (n = 802)

Fields and questions in MDH forms	Properly filled (%)			p value
	Total ships (n = 802)	Cargo ships (n = 455)	Passenger ships (n = 347)	
Port of submission	95	92	98	0.004
Date of submission	95	92	99	< 0.001
Name of ship	99	99	99	0.925
Registration/IMO number	79	68	93	< 0.001
Arriving from	96	93	99	0.005
Sailing to	68	45	97	< 0.001
Nationality/flag of ship	96	97	95	0.048
Master’s name	98	98	98	0.610
Gross tonnage	85	80	92	< 0.001
Valid Ship Sanitation Certificate	92	90	94	< 0.001
Place of issue	95	95	95	0.206
Date of Ship Sanitation Certificate	95	95	96	0.066
Re-inspection required	69	60	82	< 0.001
Has ship visited an affected area	71	60	86	< 0.001
Number of crew members on board	86	94	76	< 0.001
Number of passengers on board^a^	35	4	76	< 0.001
Completely filled nine health questions	75	63	90	< 0.001
Signed by the master	85	92	75	< 0.001
Signed by the ship’s surgeon^a^	32	0.4	73	< 0.001
Date of signature	86	85	86	0.158
Presence of Attachment to Model of MDH^b^	10	2	22	< 0.001

IMO: International Maritime Organization; MDH: Maritime Declaration of Health.

^a^ Generally only applicable to passenger ships.

^b^ The presence of an Attachment to Model of MDH indicates that there is at least one case of illness on board the vessel. Thus, this field is only applicable to ships with positive MDHs (health problem(s) on board).

As shown in Table 3, the MDH form presented at the ports did not conform to the model provided in Annex 8 in 22% of 802 ships’ MDH documents studied. In most cases, this is because a form following the model in an older Annex 8 of the IHR (2005) was submitted, for example, the model version from the IHR (1969). Furthermore, 86% of all ships had a Ship Sanitation Certificate issued from an IHR-authorised port and 89% of all ships had such certificates issued less or equal than 6 months before date of MDH. Of the 802 declarations surveyed, only 91 (11%) were positive (identifying a health problem on board); 83 from passenger ships and eight from cargo ships. Among those that were positive, seven did not fill out the Attachment to Model of MDH, which is required by the IHR (2005). Acute gastroenteritis, influenza or influenza-like illnesses, and measles were the most frequently reported health issues in the 91 positive MDH reports (45%, 10% and 10%, respectively).

**Table 3:** Proportion of passenger and cargo ships with a Maritime Declaration of Health that conforms to the model provided in Annex 8 of the IHR (2005) and reports on existence of a valid Ship Sanitation Certificate, Spain, October 2014 to March 2015 (n = 802)

Maritime Declaration of Health form	Total ships (n = 802)		Cargo ships (n = 455)		Passenger ships (n = 347)		p value
	n	Percentage of total (%)	n	Percentage of cargo (%)	n	Percentage of passenger (%)	
**Conforms to the model MDH (Annex 8 of IHR 2005)**	624	78	299	66	325	94	< 0.001
**MDH reporting a Ship Sanitation Certificate issued by an authorised port^a^**	690	86	371	81	319	92	< 0.001
**MDH reporting a Ship Sanitation Certificate issued ≤ 6 months before date of MDH**	716	89	384	84	332	96	< 0.001
**MDH positive^b^**	91	11	8	2	83	24	< 0.001

IHR: International Health Regulations; MDH: Maritime Declaration of Health.

^a^ Port included in the IHR list of ports authorised to issue Ship Sanitation Certificates.

^b^ MDH report on the presence of any health problem on board.

Some variables, including ship type, geographical area of the port, and type of MDH, were studied regarding the degree of completion of the document and validity. We found statistically significant associations between the type of ship ‘passenger’ and the following variables: (i) greater probability that the items of the MDH will be complete (Table 2); (ii) the MDH model is consistent with Annex 8 of the IHR (2005) (Table 3); (iii) the Ship Sanitation Certificate was issued by an authorised port (Table 3); and (iv) the Ship Sanitation Certificate was issued less or equal than 6 months before date of MDH) (Table 3). The comparison of positive MDHs between cargo and passenger ships could not be carried out because of the low number of positive MDHs from cargo ships. Only five of the analysed variables showed significant differences when positive and negative MDH forms were compared:

- Ship type: only 2% (8/455) of cargo ships issued a positive MDH, while 24% (83/347) of passenger ships did so (p = 0.000).
- Invalid Ship Sanitation Certificates because issued from a non-authorised port: 4% (4/91) of positive MDH forms and 15% (108/711) of negative forms (p = 0.005).
- Invalid Ship Sanitation Certificates because more than 6 months between date of issue and date on the MDH form: 3% (3/91) of positive MDH forms and 12% (83/711) of negative forms (p = 0.015).
- Missing the signature of the ship’s master: 32% (29/91) of positive MDH forms and 10% (72/711) of negative forms (p < 0.001).
- Missing the next port of destination: 13% (93/711) of the negative MDH forms and 3% (3/91) of positive forms (p < 0.000).

When comparing the MDH variables between different geographical areas we found some statistically significant differences. However, these differences disappear if the variable ‘type of ship’ is introduced in the analysis. Cargo ships are most frequently in the ports on the Atlantic Ocean coast (64% cargo ships vs 36% passenger ships) and passenger ships are most frequently in the ports on the Mediterranean Sea coast (55% passenger vs 45% cargo) (p < 0.001).

## Discussion

There are a few studies to date that analyse compliance with the IHR (2005) recommendations and the use of related documents. In 2011, Hadjichristodoulou et al. [9] reported that the MDH was the main reporting tool used for the surveillance and monitoring of communicable diseases on passenger ships in Europe. However, only 30% of the 59 competent authorities surveyed reported that they require all arriving ships to submit MDH forms. In special situations of health surveillance, as in during the early stages of pandemic influenza A in 2009, Schlaich et al. [16] showed that 32% of shipping companies experienced one or more health screening measures by port health authorities. Furthermore, a request to complete the MDH form was reported by 23% of the companies that participated in the study (7/31).

As of 2016, 115 of 171 IMO members have acceded to the FAL Convention [14]. The FAL Convention and Directive 2010/65/EU of the European Parliament and of the Council have included the MDH document among the nine documents that all ships must electronically submit (promptly and correctly filled out) if requested to do so by port authorities [11,14]. Furthermore, several studies recommend greater and better use of the MDH as modelled in Annex 8 of the IHR (2005) [17-19]. Our results show that a high proportion of the MDH forms submitted did not conform to the 2005 model (22%), have incomplete information on relevant data (only 75% completed the nine health questions) or have invalid Ship Sanitation Certificates (ca 1 in 5 cargo ships and 1 in 10 passenger ships) because the date of issue was ≥ 6 months and/or the port was not authorised. The communication of any condition on board a ship (gastrointestinal illness, haemorrhagic fevers, respiratory diseases, etc.) to port health authorities via the MDH form provides the opportunity for more efficient responses, including making a faster diagnosis, disembarking patients, identifying contacts and facilitating the adoption of health measures [20]. An incomplete MDH makes it difficult to assess risks and requires additional communication with the ship to obtain the missing information. Such omissions lengthen the response time, and reduce the effectiveness and efficiency of monitoring. This highlights the importance of fully completing the MDH form for the early detection of events and for timely implementation of public health measures on board ships, as per the WHO requirements [3].

Cargo ships require greater attention by the port health authorities as our study shows that their MDH forms are more frequently incomplete. Unlike passenger ships, freighters often lack medical services, make longer journeys and use multiple shipping routes, which can hinder the management of diseases.

Usually, when a correct and complete MDH informs the port health authorities of communicable diseases or other potential public health problems, competent authorities assess the information, confirm the situation on board and propose appropriate measures for each situation, such as isolation, landing, disinfestation, decontamination and vaccination.

It should be considered as a limitation that this study was unable to determine if all ships with potential health risks on board recorded this information on a MDH form because health authorities do not systematically inspect all ships. In addition, given that descriptive studies often represent the first scientific dip into new areas of inquiry, the limitations of this study must be addressed in subsequent analytical studies [21]. Further studies are required to assess whether the MDH is, in practice, a reliable reporting tool. For this, it would be necessary to inspect all ships arriving at ports, interview masters or doctors on board, and check whether the information recorded on MDH forms represents the reality on board; however, this is not a feasible goal.

## Conclusions and recommendations

The MDH document brings together information to assess the state of health on board a ship. Its proper completion is key to conducting a preliminary risk assessment, and efforts should be made to achieve this goal. Thus, masters and specific crew members should be properly trained to complete the MDH in such a way that port health authorities can assess the situation in advance.

We believe that an improvement in the current use of the MDH will result in improved risk assessment, greater confidence in the information provided and improve the trust between ships, health authorities and other stakeholders in the port, such as conveyance operators, relevant port authorities and surveillance system operators. Monitoring the implementation of the IHR (2005) guidelines related to the MDH should not only include monitoring the use of appropriate communication channels, but also an evaluation of the quality of the information provided. In later phases, the proper use of the MDH should be assessed and measured to prevent and provide a public health response to the international spread of disease.

Several countries might be involved in the response to an outbreak on board a ship. Therefore, the MDH should not be considered an isolated document concerning only one country, but a source of information that should be shared between port health authorities for follow-up action. The use of the SIS, a tool developed by the EU SHIPSAN project for the immediate sharing of information between ports, should be encouraged for the transmission of such information. Further, training masters on the importance of the MDH and how to routinely use it would also help facilitate prompt responses in the case of a complex public health event occurring on their ships.

Finally, further studies should be conducted to assess the real value of the MDH as a public health assessment tool for events on ships. One such avenue could be the analysis of the consequences to public health when the MDH is not properly completed.
